# Cardiovascular (Framingham) and type II diabetes (Finnish Diabetes) risk scores: a qualitative study of local knowledge of diet, physical activity and body measurements in rural Rakai, Uganda

**DOI:** 10.1186/s12889-022-14620-9

**Published:** 2022-11-29

**Authors:** Robert Ssekubugu, Fredrick Makumbi, Rocio Enriquez, Susanne R. Lagerström, Ping Teresa Yeh, Caitlin E. Kennedy, Ronald H. Gray, Lilian Negesa, David M. Serwadda, Godfrey Kigozi, Anna Mia Ekström, Helena Nordenstedt

**Affiliations:** 1 Rakai Health Sciences Program, Kalisizo, Uganda; 2 Department of Global Public Health, Karolinska Institutet, Stockholm, Sweden; 3 Department of Epidemiology and Biostatistics-School of Public Health, College of Health Sciences, Makerere University-Kampala, Kampala, Uganda; 4 Department of International Health, Johns Hopkins Bloomberg School of Public Health, Baltimore, MD USA; 5 Department of Epidemiology, Johns Hopkins Bloomberg School of Public Health, Baltimore, MD USA; 6 Department of Infectious Disease Clinic/Venhälsan, South General Hospital Stockholm, Stockholm, Sweden; 7 Department of Medicine and Infectious Diseases, Danderyd University Hospital, Stockholm, Sweden

**Keywords:** Framingham risk score, Cardiovascular, Finnish diabetes risk score, Type II diabetes, NCD 10-year risk scores, Qualitative, Uganda

## Abstract

**Background:**

Non-communicable diseases such as cardiovascular conditions and diabetes are rising in sub-Saharan Africa. Prevention strategies to mitigate non-communicable diseases include improving diet, physical activity, early diagnosis, and long-term management. Early identification of individuals at risk based on risk-score models – such as the Framingham Risk Score (FRS) for 10-year risk of cardiovascular disease and the Finnish type 2 Diabetes risk score (FINDRISC) for type 2 diabetes which are used in high-income settings – have not been well assessed in sub-Saharan Africa. The purpose of this study was to qualitatively assess local knowledge of components of these risk scores in a rural Ugandan setting.

**Methods:**

Semi-structured qualitative in-depth interviews were conducted with a purposively selected sample of 15 participants who had responded to the FRS and FINDRISC questionnaires and procedures embedded in the Rakai Community Cohort Study. Data were summarized and categorized using content analysis, with support of Atlas.ti.

**Results:**

Participants described local terms for hypertension (“*pulessa*”) and type 2 diabetes (“*sukaali*”). Most participants understood physical activity as leisure physical activity, but when probed would also include physical activity linked to routine farm work. Vegetables were typically described as "plants", “leafy greens”, and “side dish”. Vegetable and fruit consumption was described as varying seasonally, with peak availability in December after the rainy season. Participants perceived themselves to have good knowledge about their family members’ history of type 2 diabetes and hypertension.

**Conclusions:**

While most items of the FRS and FINDRISC were generally well understood, physical activity needs further clarification. It is important to consider the seasonality of fruits and vegetables, especially in rural resource-poor settings. Current risk scores will need to be locally adapted to estimate the 10-year risk of cardiovascular diseases and type 2 diabetes in this setting.

**Supplementary Information:**

The online version contains supplementary material available at 10.1186/s12889-022-14620-9.

## Background

Each year, an estimated 41 million people die from non-communicable diseases (NCDs), approximately 70% of all deaths globally; of these, approximately 17 million are among people below the age of 70 years and classified as premature deaths [1, 2]. The most common types of NCDs are cardiovascular diseases (CVD) (e.g. myocardial infarction, stroke), cancers, chronic respiratory diseases (e.g. chronic obstructive pulmonary disease. asthma), and type 2 diabetes [3, 4]. Despite these diseases being starkly different, many share the same risk factors, justifying the umbrella term of NCDs. NCDs have been increasing in low- and lower-middle income countries due to demographic changes, e.g. longer life expectancy, and lifestyle changes [5, 6]. In Uganda, cause of death statistics suggest a declining trend in infectious diseases and an increase in the relative and absolute burden of NCDs [7, 8]. A population-based survey of Ugandan adults 18–69 years regarding NCDs estimated one in four respondents had raised blood pressure; it was more common in males than females and among urban than rural residents [9]. The majority (76%) of participants with raised blood pressure were not taking any treatment to lower their blood pressure. The prevalence of raised fasting glucose including diabetes was 3.3%, and close to 90% of participants who were found to have a raised fasting glucose were not on medication nor aware of their hyperglycaemia [9]. The prevalence of hypertension among adults in our study population is estimated at 20.8% [10].

The increasing burden of NCDs in sub-Saharan Africa will strain health systems primarily designed to cater for the persistent burden of infectious diseases [11]. One approach to decrease the expected future burden on health systems is to identify individuals at risk of NCDs to implement preventive interventions to delay or prevent disease progression. Risk score models used to identify individuals at risk are common in high-income settings. The Framingham Risk Score (FRS) is a sex-sensitive algorithm used to estimate the 10-year CVD risk of an individual [12]. The FRS non-laboratory version is attractive in resource-limited settings [13, 14]. Another risk score is the Finnish type 2 Diabetes risk score (FINDRISC) [15], a simple non-invasive tool developed in a prospective cohort of individuals aged 35–64 years used to estimate 10-year risk of type 2 diabetes [16]. Such scores are increasingly used to detect risk and guide informed decision-making regarding initiation or intensification of preventive strategies but have mostly been used and validated in high-income settings [17–19]. Transferring risk scores from the setting where they were developed and tested to another type of setting can be tempting, but needs careful evaluation, calibration and ideally validation [20–23]. However, in low-resource settings, there is limited literature on the acceptability and understanding of NCD risk scoring tools and procedures.

With the increase of NCDs in sub-Saharan Africa, more knowledge about how these diseases and their risk factors are being perceived is needed. Despite the increasing risk, level of knowledge about key NCDs like stroke and their risk factors remains low [24, 25]. Several risk scores have been developed to estimate individual’s risk for acquiring one or more NCDs. Determining commonly acceptable local terms for disease, key symptoms, dietary practices, and the understanding of other key indicators is critical in assessing disease and risk. Adaptation of data capture tools is essential in enhancing rigor of measurements and data obtained for public health decision making. The choice of a tool for data collection is a key element of the research process. It is through questionnaires / instruments aimed to assess, for example, family history of disease and acceptability of performing a given procedure in each setting, that it is possible to measure a phenomena of interest and analyze their associations or risk in health surveys. As tools have been developed in the high-income countries, we were uncertain about how participants will perceive or understand some of the components of the tools and whether procedures like waist, hip measurements and others would be well accepted in a non-clinical setting.

The purpose of this study was to explore community members’ local knowledge, terminology, and understanding of key components of FRS and FINDRISC in the context of a rural population-based public health surveillance site in Uganda, using qualitative exit interviews with selected Rakai Community Cohort Study (RCCS) participants.

## Methods

### Study setting and population

The RCCS is a population-based open cohort in rural south-central Uganda from 1994 to date. Details about the study site have been published elsewhere [26–30]. Briefly, the RCCS enrolls residents and recent migrants aged 15–49 years in ~ 40 communities to participate in HIV surveillance, with additional modules and reproductive health. In 2017–2019, we introduced a question module assessing NCD risk into the RCCS, using items from the non-laboratory-based FRS and FINDRISC, as well as some key laboratory tests. The NCD module targeted participants aged 35–49 years to assess their 10-year risk for CVD and type 2 diabetes. We obtained data on age, sex, and other demographics as part of the behavioral questionnaire. Prior to the questionnaire we took blood pressure and took anthropometric measurements (height, weight, hip, and waist). We also assessed their history of type 2 diabetes and hypertension as well as current treatment and family history of type 2 diabetes and hypertension, dietary intake of fruit and vegetables and physical activity (Table 1). Quantitative results have been published previously [31].

**Table 1:** Summary of the key items from the Framingham and Finnish Diabetes risk scores and the WHO Stepwise approach introduced in the RCCS NCD module

**Key items**	Included in RCCS NCD module	Non-lab-based Framingham Risk Score	Finnish Diabetes Risk Score	WHO Stepwise Approach (core items) ^**^
Age (in completed years)	*√*	√	√	√
Sex	*√*	√	√	√
Blood pressure	√^*^	*√*		*√*
Current smoking	*√*	√		√
Body mass index (BMI)	√^*^	*√*	*√*	*√*
Waist circumference	√^*^		*√*	*√*
Current treatment for hypertension	√ ^*^	√	√	
Family history of diabetes	√^*^		*√*	
History of elevated blood glucose/diabetes	√^*^	√	*√*	
Physical activity	√^*^		*√*	*√*
Consumption of fruits/vegetables	√^*^		*√*	*√*
Alcohol consumption	*√*			√
Biochemical measurements	*√*			√

^*^Assessment data presented in this paper

^**^Not all demographic items included in the table

### Participant selection

All participants in this study were identified following their participation in the RCCS. Interviewers were trained to administer the RCCS survey including the NCD question module systems, which provided an opportunity to identify potential key informants and building rapport with them. Recruitment was done by convenience sampling. Interviewers ensured that they did not enroll more than one participant from the same household. Potential informants who were found to be knowledgeable and willing to share personal experiences with the NCD module were asked for an additional qualitative exit interview that would explore in-depth topical areas on feeding, physical activity, and perceptions on vital measurements. Written consent was obtained, and no participants were identified as illiterate. Once the respondent chose to participate, they were interviewed the same day or the following working day at the central RCCS interviewing site in the community or at another location convenient for them, while the NCD procedures and questions were still fresh.

A total of 201 interviews using the RCCS questionnaires were conducted by the three interviewers during the study period. From these, 22 potential exit qualitative interview participants were identified. Five of the non-interviewed participants could not find time on the same day or the next working day and two were not in the age eligibility bracket for the risk score. A total of 15 carefully moderated in-depth interviews were conducted until saturation was reached.

### Data collection and analysis

A team of three experienced social science qualitative interviewers (1 male, 2 females) were trained to administer the RCCS survey including the NCD risk assessment module. The interviewers received training on the rationale for each of the items on the risk scores and how the items are reflected on the qualitative semi-structured interview guide to probe responses and gain a deeper understanding of how participants interpreted the global tools and the thought process behind their responses.

Between January and April 2019, interviewers recruited and interviewed RCCS participants per the participant selection procedures above. A semi-structured interview guide was developed in English by the first (RS, MSPH coordinator of the RCCS activities) and the last authors (HN, MD, PhD co-investigator to the NCD risk assessment protocol) and later translated into Luganda the predominant local language. The translation was done by a professional Luganda teacher who is attached to the RSHP data quality control team. Key items on the FRS and FINDRISC were considered while developing this study interview guide (Additional file 1). The study guide was pilot tested in five people.

Interviews were audio-recorded and transcribed by the interviewer within 48 h, and field brief notes were made during the interview. Interviews last an average of 35 min. After each interviewer had collected and transcribed their first interview, we had a review meeting with the interviewing team to brainstorm on their experiences and to determine how to proceed with subsequent data collection. All the interviews were held in the local dialect (*Luganda*) but translated to English directly at transcription. After transcription, a second team member read through the transcript as they listened to its audio recording and read its field notes to ensure completeness of transcription and obtain common agreement on translation between the transcribing interviewer and the second reader.

We used thematic analysis approach to analyze the data. The analysis commenced iteratively with the collection process. During the collection process we discussed emerging and diverging themes and decided on how to proceed with the inquiry especially on how to improve probes. The scripts were read numerous times by RS and LN to identify patterns in the data and develop potential codes. Transcripts were coded in Atlas.ti version 5.2 using short-listed preliminary codes. Using “lean coding” [32], the initial 6 codes expanded to 22 codes as more themes emerged. After overlapping and redundant codes were merged or dropped respectively, 19 codes remained and were recategorized into domains. The findings are reported below in anonymized quotes that were translated into English from the verbatim interview script.

### Ethics consideration

Each informant in this sub-study provided written informed consent for RCCS participation, including the NCD module, and a separate consent for the additional qualitative exit interview. The study went through institutional ethics approvals at the Uganda Virus Research Institute’s Research Ethics Committee (GC\127\18\07\657), the National Research Registration by the Uganda National Council for Science and Technology (SS 4836) and the Swedish Ethical Review Authority (2018\2542–31\2). The interviewers had human subjects research ethics training within the past three years prior to their involvement in the study. No study participants' names were used in the final coded transcripts.

## Results

A total of 15 interviews were conducted with 9 women and 6 men. The median age was 44 years (range 35–49) for men and 37 (range 35–45) for women. Participants’ occupations were substance agriculture/farming (4) (small scale farmers producing largely for their home use), trader/vendor/shopkeepers (5), fisherfolk (2), government/clerical workers (2), restaurant/waiter (1) and a housewife (1). They were mainly household heads (8) and spouses of household heads (6), or held another relationship with the household head (1). Key results are summarised with sample codes and illustrative quotes in Table 2.

**Table 2:** Sample codes and selected quotes

Theme	Sample code(s)	Illustrative quotes
Local term for hypertension	Pressure "*puleesa*" Pulse *“entunuunsi”*	… it is *puleesa* [pressure], that’s what I hear people call it… [Participant 13, Female, 42 years old, Government/clerical worker] people understand but the elderly tend to also call it *entunnunsi* [pulse]. I hear people in this area say that I have palpitation [pulse] [Participant 1, Female, 37 years old, Farmer]
Local term for type 2 diabetes	Sugar "*Sukaali*"	*Sukaali*… I am told the sugar is a lot in their body, so we call it *sukaali* [sugar] [Participant 3, Female, 35 years old, Agriculture]
Local term for NCDs	Diseases of the rich Diseases of the elderly	They usually call them diseases for the rich people… in the past they [the diseases] used to attack fat people who are rich [Participant 10, Male, 41 years old, Farmer] Yes, I always hear them calling them diseases for the elderly persons. I have never heard of a term for them. [Participant 11, Male, 46 years old, Trader]
Experience with vital measurements	Comfortable/usual medical procedure	Nothing that I felt [comfortable] – a health worker can do anything to check your body, you need it for your good health. [Participant 13, Female, 42 years old, Government/clerical worker]
Physical activity	Games (mainly football mentioned/youths)	Among all people including the children, youths, elderly, and women, I only see youths playing football. I have never seen elderly persons jogging, I have never seen them playing football, … [Participant 10, Male, 41 years old, Farmer]
	Manual labour Digging/brickmaking & Others	Most people cultivate because it is the usual activity to get food but not with intentions of having a healthy body. Even those one making bricks, they do it to get money but then find themselves with healthy bodies hence beating two birds with one stone.. [Participant 15, Male, 38 years old, Fisherman]
Measurement of physical activities	Hours spent/level of engagement	It usually takes me five hours…that is I usually go to the garden at 7:00am and end cultivation/digging at 12:00 pm [Participant 10, Male, 44 years old, Farmer]
Fruits and Vegetables	Common examples Local knowledge / seasonality	Mangoes, watermelons, and pineapples I eat them [fruits] when on season from December to end of February… I eat like five a day apart from pineapples mainly in July [Participant 12, Female, 37 years old, Housewife]
	Local term(s) for vegetables Examples	*Enva endiirwa*, (side dish), leafy, greens *ddoodo* [a green colour type of amaranthus spinach]*, nakatti* [scarlet eggplant]*, bbuga* [a purple-coloured type of amaranthus spinach]*, ejjobyo* [African spider herb] [Participant 7, Female, 38 years old, Trader/vendor]
Knowledge on family history	Awareness of family member with hypertension or type 2 diabetes Reason to know obtain assistance	If there was any of them who had been diagnosed with diabetes, they would have told me but no one has ever said a thing about being diagnosed with diabetes however I had a cousin (paternal aunt’s daughter) who is diabetic and I used see her injecting herself and later she told us… People say that when you use herbs, you can get healed of diabetes, […] that could be one of the reasons people share this information as compared to HIV/ AIDS which has no cure. Participant 7, Female, 38 years old, Trader/vendor

### Terms and local knowledge about type 2 diabetes, hypertension, and NCDs

We explored the commonly used local terms for type 2 diabetes, hypertension, and NCDs in general. In the exit interviews, we used the terms “*sukaali*”, which also means sugar, for type 2 diabetes and “*pressure”* for hypertension. All the participants agreed to and concurred with the use of the term “*sukaali*” for diabetes.

Informant: *I do not know of any other word in Luganda, it is sukaali in Luganda we call it sukaali* [literally meaning sugar]*. Is there any other name in Luganda* [informant asks]*?*

Interviewer: *I don’t know, I am here to learn from you.*

Informant: *Another name for diabetes? No madam, I have never heard of any other term.* [Participant 14, Female, 35 years old, Farmer]

All but one participant exclusively used the term “*pulessa"* [pressure] for hypertension, and one participant also introduced the term “*entunnunsi*” [pulse, palpitation].

*Blood pressure in this community has been localized. It remains [puleesa] pressure you say pressure, people understand but the elderly tend to also call it [entunnunsi]* [palpitation]. *I hear people in this area say that I have palpitation, or that they have told me that I have ...pressure, that is how it is in our area.* [Participant 1, Female, 37 years old, Farmer]

We explored if there were local terms that could be used generically to mean non-communicable/ non infectious diseases. Three participants suggested the use of the term “*endwaddwe z’abakadde*” [diseases of the elderly]. It was common for informants to associate diabetes with age and abnormal weight; however, hypertension was mainly linked to being wealthy “*abaggaga”* [the rich]. As one participant indicated:

*For debates [diabetes] I always hear people say that it attacks elderly people and those with abnormal weights and for hypertension like I told you it attacks rich people.* [Participant 10, Male, 41 years old, Farmer]

We probed for this at subsequent interviews, but subsequently participants expressed discomfort with the use of diseases of the elderly to refer to non-communicable, non-infectious diseases. One participant indicated that “*endwadde z’abakadde*” is used for musculoskeletal diseases like back pain but not the classical NCDs:

*Diseases for the elderly people here it is usually used when referring to diseases concerning bones and may be the back pain.* [Participant 15, Male, 38 years old, Fisherman]

A few participants were quick to refute the notion of “diseases of the elderly”, noting that recently these diseases have become more common, and they affect people of all ages, so referring to them as diseases of the elderly was anachronistic:

*Those diseases like pulessa* [pressure] *and sukaali* [diabetes] *are now very common diseases that they do not vary by age that is, they cut across all ages not only the older people.* [Participant 2, Male, 38 years old, Government/clerical worker]

### Experience with anthropometric measurements

We explored the acceptability of taking anthropometric measurements like waist and hip circumference, height, and weight in a non-clinical setting, probing especially for acceptability and experience with hip and waist measurements and whether it was appropriate for a research assistant to take the measurements of a participant of the opposite sex.

Most participants presented no sociocultural hinderances for male research assistants to take measurements of female participants and vice versa. Two participants felt that this was a medical procedure where the sex of your provider does not matter. Others equated this to going to a tailor who must measure your height, waist, etc. as stated by this participant.

*We take it as a usual thing because when you take your clothes to a tailor, he/she will take your measurements, to do a good outfit. But this which is done by you people the basawo [health workers], I don’t know unless you tell me of any problem.* [Participant 1, Female, 37 years old, Farmer]

A few participants, however, felt this required more explaining, especially if the person taking their measurements was of a different sex. Some said they would be “skeptical as to why it was a man.” This concern was more likely raised by female participants:

Participant: I did not feel anything because it is a female who took my measurements. If it were the opposite sex, I would be skeptical.

Interviewer: If it were an opposite sex, how would it be, how would you feel?

Participant: It would all depend on how he would handle me and may be his approach too.

Interviewer: Please throw more light on that?

Participant: If he comes and explains to me the whole process, it is ok, but just bumping on me, that will mean something different. I would become skeptical.

Interviewer: You say you would be skeptical, why and what would be your reaction?

*Participant: I would ask questions like, why take the measurements of my waist and why would it be he* [him]*?* [Participant 12, Female, 37 years old, Housewife]

Participants were asked about their feelings about having their blood pressure taken and recorded. Almost all participants indicated that this was acceptable. However, a few reported being anxious about what the reading would be and sometimes the reading was indeed frightening.

*I felt so good. I was not forced to come here. I only got scared when they told me that my blood pressure was high, I didn’t know about it, but with the rest, there is no problem.”* [Participant 7, Female, 38 years old, Trader/vendor]

### Terms, local knowledge, and experience with physical activity

We explored the understanding of physical activity in this rural population. We asked what physical activities participants were involved in (if any) and what physical activities were commonly done by other community members. For purposes of the interview, we formally translated physical activity as “*duyiro*”, which informants more often perceived to mean “exercising”, though the terms could sometimes be used interchangeably.

About half of the participants did not immediately understand the question(s) on physical activity. Oftentimes interviewers had to re-ask, re-phrase or even paraphrase the question, and sometimes had to use illustrations like "activities of sports nature" or "activities that cause you to breathe hard or pant". Participants mentioned a variety of events that they considered physical activities. Most mentioned energy exerting or activities of daily living, like housework (paid or unpaid) especially working in the garden (mainly digging and cutting grass), grazing animals and house chores like washing clothes, and fetching water, rather than mentioning recreational physical activities.

Interviewer: Okay, you say you have not been putting attention to it. Let’s try to understand this together. Tell me about the exercising or manual thing you do?

*Informant:Cultivation, you know we farmers are in the garden and when I dig, sometimes in the morning and return in the evening it is enough exercise.* [Participant 1, Female, 37 years old, Farmer]

This was similar to what participants observed in their communities as the most common forms of physical activities:

“*The activity I see mainly is farming* [digging] *and here they do activities like spraying and that spraying can is so heavy and needs one to be strong because it is really tiring*”. [Participant 9, Female, 41 years old, Farmer]

Some participants did not consider incidental physical activities as exercise. Although structured recreational physical activities were rare, football was commonly mentioned in addition to daily manual work activities. Almost all the participants who raised it associated it with youths, and none of the informants indicated that they played football themselves.

Interviewer: What types of physical activities do people in your community engage in?

Informant: The youths mainly get involved in playing football and cultivating

Interviewer: Apart from cultivating and playing football, tell me about the other physical exercises do they get involved in?

*Informant: Apart from those, there no other physical exercises that they are involved.* [Participant 5, Male, 49 years old, Shopkeeper]

### Measuring physical activity

We asked participants to describe the amount of time they spent engaged in different types of physical activity. Among those who engaged in physical activity, the most frequently reported amount of time spent in the field digging gardening was 5–6 h each day, either in one go or combining morning and evening hours of cultivating, 5–6 days each week:

*When it’s cultivation time, I wake up at exactly 06:00am, wash my face, serve the pigs food then make sure that at exactly 07:00am I am already in the garden cultivating, then I get off cultivating at 12:00pm which I always do six days in a week.* [Participant 10, Male, 44 years old, Farmer]

Other activities generally took less time, but those which were quantifiable still took at least one hour. Two participants were manual laundry ladies; they considered their work as their physical activity as described by this participant.

*I will tell you that I wake up at 7:00am to start washing for clients and by 10:00am am done with this, then I continue with ironing and by noon am done with the ironing, lunch is usually prepared by my big sister, after washing, I take a shower, have lunch then take a nap.* [Participant 7, Female, 38 years old, Trader/vendor]

The frequency of the most popular incidental physical activities varied by season. Community members were likely to be more physically engaged during the rainy season than during the dry seasons:

Interviewer: You also talked of cultivation, do you this only in the morning or you sometimes do it in the evening?

*Participant: This varies, during dry seasons, we only dig in the morning and rainy seasons, both morning and evening.* [Participant 1, Female, 37 years old, Farmer]

### Terms and local knowledge about fruits and vegetables

We assessed key informants' understanding of vegetables and solicited examples of vegetables that were part of their diet (if any). We posed a question to the informant: “In your just concluded interview, the interviewer asked you to talk about vegetables. What did you understand by vegetables [*enva endiirwa*]? What are they and how did that discussion go?".

Some informants mainly described vegetables as “*bikoola*” [leafy], green in colour [*greens*] (mentioned in more than half the interviews), or side dish [*enva endiirwa*] (consistent with the translation we had used in the questionnaire, "bitter or sour leaves"). Sometimes, informants emphasised their understanding of vegetables by mode of preparation, or the state in which they are served like “*oyinza okuzirya embisi nga cabbage*” [eaten raw like cabbages], half cooked or cooked fully, steamed to taste, or fried in oil. It was more common for informants to use illustrative description through which they referred to specific or common vegetables, sometimes considered as wild plants. The informant below provided a detailed list of different vegetables. which description has important connotation on how vegetables may or may not be valued in this type of setting.

Interviewer: I would like to know what you understood when we asked you about vegetables.

*Informant:* *What I understand from vegetables the greens we grow, and some is wild like *ddoodo [a green colour type of amaranthus spinach]*, *nakatti [scarlet eggplant*], *bbuga [a purple-coloured type of amaranthus spinach]*), *ejjobyo [African spider herb] *which is served as enva endiirwa* [as a side dish]. [Participant 7, Female, 38 years old, Trader/vendor]

This informant ends with the term “*enva endiirwa*” [side dish], a description that has importance in respect to the way vegetables are perceived, as indicated by another informant:

*As you hear, it is a side dish … you can have it if you have it, it is of less importance when you do not have it… you would be lucky to have the real sauce beans, meat [laughs].* [Participant 5, Male, 49 years old, Shopkeeper]

Participants also described the advantages of eating vegetables, indicating that they are foods that prevent against diseases through cleansing of the body, increasing blood supply in the body and keeping one generally healthy:

*I know of vegetables as something that adds to our health, they help in okuyonja omubiri [*detoxification?*] and prevents diseases from attacking you…, when you lack blood, the health workers advise you to take the vegetables and get blood there are in many categories with different uses* [advantages] [Participant 3, Female, 35 years old, Farmer]

Informants also described their understanding by combining mode of preparation and usefulness of the vegetables especially in fighting disease. They used terms like eating them raw, half cooked, fried, steamed; when needed, we probed for how the different vegetables in their communities were prepared or served. As this participant stated:

*There are those we eat raw, half cooked, and others fried. Most times, the ones we eat raw or half cooked help in the fight against diseases as compared to our counterparts* [other people in the community] *who fry them. Vegetables lose nutrients depending in the way they are handled. For example, Ddoodo, when cut them before washing, all the nutrients are lost in the process and there also people who cut and fry them, these eat roughage. If it is cabbages, it is better when eaten raw so that the body gets all the value in it that is if you know how to prepare them well and hygienically.* [Participant 1, Female, 37 years old, Farmer]

Participants often used specific examples to illustrate their knowledge of fruits. “*miyembe*” (mangoes), and “*ffene”* (jackfruit) were the most common examples participants highlighted. Other participants described them as edibles that give energy or add blood and water to the body:

*Fruits give energy and there are those that add blood and water to the body… for example we have passion fruits, paw paws [papaya], mangoes, watermelon, and orange.* [Participant 13, Female, 42 years old, Government/clerical worker]

It was common for participants to describe fruit as something picked from the tree, eaten raw [uncooked], or can be eaten without the efforts of preparation.

Some informants linked fruit consumption more to children than to adults. They indicated that children may survive on fruits the whole day, but adults typically took fruits "by the way" and could spend longer periods not eating fruits:

*I can even take a month without eating it again, but this does not apply to other members in the community especially the younger ones. There are guys in the community who survive on jackfruit. They hide the jackfruit bunches and eat them when they are ready. Those are most especially children – but for an adult, no [laughs].* [Participant 2, Male, 38 years old, Government/clerical worker]

Participants emphasized that unless one has multiple trees of a given fruit, they would not generally be willing to sell the fruits. Instead, they would leave the fruits for the children, as this informant indicated:

*If someone has one tree in the compound, there is no way they can sell them, instead, they leave them for the children.* [Participant 1, Female, 37 years old, Farmer]

When talking about fruit and vegetable intake, it was common for informants to refer to seasons when fruits and vegetables were less or more available. It was not uncommon for participants to refer to abundant access and scarcity, linked to annual seasons, as well as “bad year” to mean years of low harvest (even within the seasons of a given fruit or vegetable):

*We have various fruits in our community like, avocado, paw paws [papaya], jackfruits, oranges, tangerines, mangoes, watermelon. Though these are seasonal and current, they are not available in the community – difficult to get them.* [Participant 7, Female, 38 years old, Trader/vendor]

Seasonality as a factor of access and intake was also mentioned in respect to vegetables. However, unlike fruits, most vegetables were available almost throughout the year though in varying quantities:

*We usually have some throughout the year especially Nakatti and Ebbuga because they are planted in swampy areas during the dry seasons and on mainland during rainy seasons… then we buy from those who have swampy areas. This is different with ddoodo, during dry seasons, there will be no ddoodo at all because it is not planted in swamps like the other vegetables.* [Participant 12, Female, 37 years old, Housewife]

### Knowledge on family history of type 2 diabetes and hypertension

Informants were asked if and how they were able to respond to questions on family history of type 2 diabetes and hypertension. Most informants perceived themselves to have a good level of knowledge about the health of their close family members, even if they were not currently living with them. A few informants felt that some diseases would be treated privately, so would not be disclosed even within family, but diabetes and hypertension were not those types of diseases in the private domain:

*Informant: There are diseases you cannot tell people if you have them for instance candida, syphilis but not for diabetes, why not share, you never know among those you tell, there could be one who can give you a permanent solution.*

Interviewer: What do you think makes people not to tell others that they have candida and syphilis?

*Informant: It is because these diseases are of the private parts, you can’t stand there and tell people that you know what, my private parts are itching or paining. If you do that people will think that you are mad. But for diabetes, even if it is1000 people, I can tell them that I have diabetes and ask if they can be of help.* [… even with hypertension] *There is no problem with that. In fact, in our community, the term (pressure) has been locally adopted. People are so free with that information.* [Participant 7, Female, 38 years old, Trader/vendor]

Some informants felt that close relatives, mainly siblings and parents, would disclose any conditions for which they received a diagnosis:

*As I told you my siblings would open to me in case of such a condition, if my elder sister would be open about her HIV status to me and my other siblings, then there is no reason she or? any of them would keep quiet about such a thing like diabetes.* [Participant 1, Female, 37 years old, Farmer]

## Discussion

With the increase of NCDs in sub-Saharan Africa, more knowledge about how these diseases and their risk factors are being perceived is needed. Determining commonly acceptable local terms for disease, key symptoms, dietary practices, and the understanding of other key indicators is critical in assessing disease and risk. In our study, hypertension and diabetes were generally well understood. The most common term in our setting to denote hypertension was *pulessa* [pressure]. The common local term for type 2 diabetes was *sukaali* [literally translates as sugar]. Both terms were widely accepted. This solid understanding of the terminology made the data gathering process easier. However, like in many other settings, there is not one accepted umbrella term for NCDs which makes communication about these diseases in general terms complex. From a broader perspective, informants identified the conditions as “*diseases for the rich*”, as was the case historically when they were associated with economic development in high-income countries [33]. They were also referred to as “*diseases for the fat people*”; multiple studies have confirmed that overweight and obesity are indeed risk factors for many NCDs [4]. Some referred to them as “*diseases for the elderly”* as has been historically known that older people have a an increased risk for different NCDs relative to younger counterparts. In summary, the population identifies these diseases by the risk factors like older age, being obese or overweight and being wealthy.

Waist circumference has been shown to be more informative in predicting raised blood pressure (BP), glucose and total cholesterol than body mass index (BMI), also in low-resource settings [34]. Yet unlike other anthropometric measurements that are more commonly taken waist circumference is rarely measured [35]. Health workers are concerned that patients will be embarrassed about waist circumference yet patients rarely report this [36]. At the start of this study, our field teams worried that participants would be embarrassed, but in general our participants did not express any form of embarrassment. Other than two participants who suggested that waist measurements should be taken by a same-sex health worker, most participants considered waist circumference a normal procedure that is acceptable if required by their health provider. This high level of acceptability, with rare exceptions, is consistent with what has been reported elsewhere [36].

Participants did not spontaneously respond affirmatively to questions on physical activity of a leisure or sporting nature. It was after they were probed for manual housework or farm work that informants listed a range of non-leisure physical activities, mainly citing digging, walking and other manual work within and outside the home setting. Digging and raking have been studied and categorized as “high-intensity” physical activities previously [37], and were also described as such by some of our participants as illustrated by quotes above. Community members in Uganda generally spend a lot of time in the fields/garden and are often engaged in other manual activities, which could explain the minimal attention given to leisure-related physical activities despite relative high levels of physical activity compared to other populations globally [38]. Understanding the nature of physical activities in a setting is essential in building a comprehensive, meaningful and locally appropriate physical activities index [39].

Family history has for long been known to have a strong association with the risk for type 2 diabetes and hypertension [40–42]. However, sometimes health providers have expressed challenges in obtaining reliable and accurate information on family history from their patients [43]. Our study revealed that participants were not having any difficulties obtaining information about familial history for chronic diseases, where there was no stigma attached. Mainly through regular information sharing with family members, informants considered themselves as a reliable source of information about their family history for chronic diseases.

Participants were generally in agreement about what vegetables are, describing them as leafy and/or plants. They identified vegetables as side-dishes eaten as a compliment to the main meal eaten fresh and cooked in different forms. Precisely, fruits were mainly defined as food stuffs that grow on trees with flowering characteristics like mangoes and some climbing plants like passion fruits, watermelons. The most commonly mentioned fruits were mangoes, pineapples, watermelon, and passion fruits. This list is consistent what has been described by others especially in the field of agricultural value addition [44]. The consumption of fruits and vegetables is associated with seasonality of abundance and scarcity. Most fruits are only available once or twice a year and around months immediately after the rainy seasons. While availability of vegetables tends to cluster around the rainy seasons, a few are available throughout the year supplied by farmers in swampy areas. The seasonal availability of fruits and vegetables could in part explain the reported low consumption in most parts of Uganda [44, 45]. Additionally, the seasonal availability and geographical location could influence how participants respond to questions on fruits and vegetables thus impacting the risk score measurements (the distribution of rains, fruits and vegetables is heterogenous even within the same country).

Being able to understand elements on risk score is important as this enhances future risk assessment. However, future studies to describe the understanding of risk is essential. A previous study has highlighted the complexities in understanding the concept to risk for CVD in an African setting and this requires further interrogation [46].

Like all other research, this study has strength and limitations. The primary strength of the present study is that this is one of the first to qualitatively explore participants’ experiences with Framingham and FINDRISC 10-year risk scores in a resource limited setting. The study is conducted within a unique setting of a population-based prospective surveillance cohort; this gives the opportunity for proper integration of NCD risk assessment within a public health surveillance program.

However, it is not without limitations. The studied population comprised of 35–49-year-olds based in rural south-central Uganda. This constraint is dictated by the ongoing parent study from which this sample was obtained and therefore limits our ability to infer the results to older age groups or other settings. Additionally, the semi-structed interview guide was translated into Luganda, yet the study purposed to explore, among other things, the proper local terms. To address this, our interviewers used multiple techniques to probe and assess whether the pre-translated terms were the most appropriate and if there were other popular terms. Finally, we did not have a good opportunity to triangulate our data collection methods; for instance, focus group discussions could have been conducted to explore more general norms, but we deemed this methodology unfeasible to assess individuals’ experiences immediately following their RCCS interview and procedures.

## Conclusions

This study found that terms used in NCD risk factor surveys varied in their acceptability among respondents in a population-based cohort study. For hypertension and type 2 diabetes there are commonly acceptable local terms but not for NCDs as an umbrella term. The use of disease-specific local terms may be more appropriate than use of the NCD as an umbrella term in areas where there is no agreed local term. Physical activity is mainly defined in terms of daily routine or manual work, but notably, most participants did not count non-leisure physical activity when simply asked if they engage in physical activity. Consumption of fruits and vegetables is affected by seasons of availability and scarcity. While the risk scores are generally suitable, it is important to localize key aspects, especially physical activity and taking seasonality into account for fruit and vegetable consumption. Engaging communities prior to data collection to obtain contextual knowledge on nature and load of work and other manual events could help improve studies that aim to score risk for NCDs in different settings, especially when using risk scores developed in other settings. However, in the future, locally developed and validated risk scores taking these aspects into account would be ideal.

## Supplementary Information

**Additional file 1.**

## Data Availability

Data beyond what is presented in this manuscript is available upon reasonable request to the corresponding author.

## Abbreviations

BMI: Body Mass Index
BP: Blood pressure
CVD: Cardiovascular disease
FINDRISC: Finnish Diabetes Risk Score
FRS: Framingham Risk Score
NCD: Non communicable diseases
RCCS: Rakai Community Cohort Study
