# Supplementary material for: Cardiovascular (Framingham) and type II diabetes (Finnish Diabetes) risk scores: a qualitative study of local knowledge of diet, physical activity and body measurements in rural Rakai, Uganda
Source: BMC Public Health. 2022 Nov 29;22:2214. doi: 10.1186/s12889-022-14620-9 (PMC9706863; doi:10.1186/s12889-022-14620-9)
Supplement: Supplementary file 1 — Additional file 1. [file 12889_2022_14620_MOESM1_ESM.doc]

# Key Informant In-Depth Interview Guide

**Behavioral Component of the Non-Communicable Diseases Risk Assessment study (Framingham and Findrisc)**

**INTRODUCTION**

Our purpose in talking with you today is to discuss the non-communicable diseases risk assessment and the questions we asked you about your dietary choices including use of fruits and vegetables, your physical activeness, your personal and family health history and use of medications for diabetes (type II) and hypertension. You are going to be educating me on what is going on in your community and in your personal life, the foods you and other people eat, the physical activities you engage in and other matters relating to your risk for non-communicable diseases.

**Dietary practices and food choices**

- What are the main types of foods, fruits and vegetables that people in your community eat?

(Interviewer list all the commonly mentioned foods mentioned, work with the participant to classify the foods into three (i) vegetables (ii) fruits (iii) other foods

- What types of foods are more likely to be eaten by elderly people (over 50 year olds), adults (20-50), adolescents and youths and infants?

(Interviewer probe to establish whether there are differences in the types of foods eaten at different age brackets)

- What types of foods are more likely to be eaten be elderly people (over 50 year olds), adults (20-50), adolescents and youths and infants?

(Interviewer probe to establish whether there are differences in the types of foods eaten at different age brackets)

- How do people serve the fruits? How do you count the fruits or servings of a fruits? What is a serving?
- What types of foods that are eaten by men and not women or vice versa? Why is this so?

Probe whether this is customary or by choice?

- What are the different types of foods that people eat / are available in your community over the different seasons of the year?

Describe the seasons and the types of foods that are available or not available

- Seek for what seasons have fruits and vegetables and in which seasons they are lacking?
- How many days in a week do you eat fruits? How have you counted the days, what did you consider?
- How many times in a day do you eat a fruit? How have you counted the days, what did you consider?

**Physical activity**

- What types of physical activities do people in this community engage in?
- What types of activities are engaged by the different types of people

Probe for (men, women older or younger people)

For how long do you perform the physical activities – how do you determine the time spent? Does the time differ across the time span? How and in what ways

- What type physical work do people in your community get engaged in?

Non-intentional physical activities)

- Have you ever been diagnosed with Diabetes?

If yes, is there any this you’re doing about your diabetic condition?

- Have you ever been diagnosed with hypertension?

If yes, is there any thing you’re doing about your diabetic condition?

- Is it possible for you to know if any of your family members has been ever diagnosed with Diabetes or Hypertension?

If yes: How would you know about this? What kind of family member would share this information?

If no: why wouldn’t you know about such important information?

Are there family members that would not share this information? If yes, why?

- Has anyone in your family ever been diagnosed with Diabetes?

If yes, is there any this they are doing about your diabetic condition?

- Has anyone in your family ever been diagnosed with hypertension?

If yes, is there any they are doing about your hypertensive condition?

- When we talk of diabetes what do you think this condition means? (signs, symptoms – local knowledge)
- When we talk of hypertension what do you think this condition means? (signs, symptoms – local knowledge)

Ending: We had discussed a number of issues (give a summary of the key issues that came up in the discussion) is there anything else you would like to say?

Concluding remarks

Thank you for accepting to talk to me today, we may come back to you if you accept to clarify the information you have provided. The information you have provided will be very useful in helping us determine how well to assess one’s risk for non-communicable diseases.
